# Systemic treatment for lung carcinoids: from bench to bedside

**DOI:** 10.1186/s40169-019-0238-5

**Published:** 2019-07-04

**Authors:** Mariangela Torniai, Laura Scortichini, Francesca Tronconi, Corrado Rubini, Francesca Morgese, Silvia Rinaldi, Paola Mazzanti, Rossana Berardi

**Affiliations:** 1 Clinica Oncologica, Università Politecnica delle Marche, AOU Ospedali Riuniti di Ancona, Via Conca 71, 60126 Ancona, Italy; 2 Section of Pathological Anatomy and Histopathology, Department of Neuroscience, Università Politecnica delle Marche, AOU Ospedali Riuniti di Ancona, Ancona, Italy

**Keywords:** Carcinoids, Neuroendocrine, Lung, Somatostatin analogs, PRRT, Everolimus, Chemotherapy

## Abstract

In the huge spectrum of lung neuroendocrine neoplasms, typical and atypical carcinoids should be considered as a separate biological entity from poorly differentiated forms, harboring peculiar molecular alterations. Despite their indolent behavior, lung carcinoids correlate with a worse survival. To date, only limited therapeutic options are available and novel drugs are strongly needed. In this work, we extensively reviewed scientific literature exploring available therapeutic options, new molecular targets and future perspectives in the management of well differentiated neoplasms of bronchopulmonary tree. Systemic therapy represents the main option in advanced and unresectable disease; accepted choices are somatostatin analogs, peptide receptor radionuclide therapy, everolimus and chemotherapy. To date, an univocal treatment strategy has not been identified yet, thus tailored therapeutic algorithms should consider treatment efficacy as well as safety profiles. Several molecular alterations found in carcinoid tumors might act as molecular targets leading to development of new therapeutic options. Further studies are necessary to identify new potential “druggable” molecular targets in the selected subset of low-grade lung carcinoids. Furthermore, evaluating the available therapies in more homogeneous population might improve their efficacy through a perfect tailoring of treatment options.

## Background

Neuroendocrine neoplasms (NENs) include all tumors derived from the diffuse endocrine system. They can arise everywhere, since neuroendocrine cells are virtually distributed in each organ. The most important site for incidence is gastrointestinal tract; then we can number bronchopulmonary tree.

In the huge spectrum of lung NENs, the most important distinction split between well differentiated and poorly differentiated neoplasms. The first group includes typical carcinoids (TCs) and atypical carcinoids (ACs), characterized by a low mitotic count with absence of necrosis. In particular, TCs have fewer than 2 mitoses/2 mm^2^ and no necrosis, whilst ACs show 2–10 mitoses/2 mm^2^ and/or foci of punctate necrosis. The second group includes large cell neuroendocrine carcinoma (LCNEC) and small cell lung cancer (SCLC). They both have more than 10 mitoses/2 mm^2^ and extensive necrosis. Others cytological criteria are needed to differentiate LCNEC and SCLC as dimension of tumor cells, prominence of nucleoli and chromatin pattern [1–3]. Although persistent debates about grading system [4], a classification based on Ki-67 index could be useful for patient’s stratification and prediction of prognosis. This is especially true for carcinoids, even because there is a small core of well differentiated NENs with a relatively high proliferative index showing the ability to metastasize as poorly differentiated forms [5]. Therefore, Ki-67 might act as a prognostic factor also in lung NENs, even if some authors have shown its limited utility in predicting survival when used alone [3]. In a recent study, Rindi et al. suggested the use of Ki-67 index and mitotic count in combination with histological characteristics developing a new grading system (G1–G3) [6]. The WHO 2015 classification of lung tumors represents the current classification system of lung NENs including both morphological characteristics and proliferation index; mixed forms consisting of a combination of poorly differentiated NENs (SCLC and LCNEC) with conventional squamous cell or adeno-carcinoma component are also included [7].

The introduction of next generation sequencing (NGS) techniques has provided a big boost in the knowledge of cancer molecular landscape. Unfortunately, the application of NGS in lung carcinoids still remains poor if compared to other lung tumors, maybe owing to the rarity of the disease as well as the lack of knowledge of these techniques in this setting [8–12].

Fernandez-Cuesta et al. sequenced the whole exome of 69 pulmonary carcinoids reporting the most frequently DNA alterations found in these neoplasms. Well differentiated NENs frequently harbor alterations of chromatin remodeling genes, especially MEN1, PSIP1 and ARID1A [9]. MEN1 maps on chromosome 11 and encodes for menina, a nuclear protein involved in cell replication, DNA repair and transcription process. Mutation or loss of this gene can be found in 13% of sporadic pulmonary carcinoids and seem to be associated to shorter survival [13]. Furthermore, almost 5% of patients with lung carcinoids have MEN1 syndrome harboring a germinal mutation of this gene; in these cases, family history, clinical examination and laboratory tests like calcium, parathyroid hormone (PTH) and prolactin should be performed as well as a screening for mutational analysis [14–16]. Additionally, alterations of histone methyltransferase are frequently discovered in well differentiated lung NENs by mutations of the polycomb complex (CBX6) and EZH2; [17] adding aberrations of histone modifiers BRWD3 and HDAC5, at least 40% of lung carcinoids seem to harbor mutations in genes involved in histone modifications [9]. At the opposite side, mutations of cell cycle checkpoints and cell differentiation regulators represent the most common aberrations in LCNEC and SCLC [18], whilst RB1 and TP53 mutations are uncommon in lung carcinoids, with a little more prevalence in ACs than in TCs [19].

Moreover, the strong association with smoking could explain why poorly differentiated NENs exhibit a higher somatic mutation rate (> 7 per million base pairs) compared with TCs and ACs (< 1 per million base pairs) [11]. On this basis, TCs and ACs might be considered a separate biological entity from LCNEC and SCLC and not a continuum of diseases [9].

Lung NENs represent 25% of all primary lung tumors [20] and approximately 25% of all the NENs of the human body [21, 22]. In the last decade, an increase in their incidence was shown probably due to a gain in diagnostic tools (most of all histology and immunohistochemistry). The incidence of lung NENs is reported to be up to 1.57/1,000,000/year; [23, 24] SCLC represents the most common histotype (15% of invasive primary lung cancers), followed by LCNEC accounting for approximately 8% of lung tumors; bronchial TCs and ACs represent the rarest entity (1–2% of all lung cancers and 8% of lung NENs respectively) [25].

Patients with lung carcinoids are usually younger than patients with poorly differentiated NENs (median age 48 vs 70 years old) with a slightly difference between TCs and ACs regarding the age of onset (45–50 years for TCs and 59 years for ACs) [26–28].

Clinical presentation of lung carcinoids is related to their localization rather than tumor differentiation: central neoplasms often cause symptoms related to airway obstruction (cough, recurrent pneumonia, chest pain, wheeze and dyspnea) simulating infections or asthma, whilst peripheral carcinoids are often asymptomatic and incidentally diagnosed [29].

Carcinoid syndrome with the risk of cardiac right valve disease rarely appears in patients with bronchopulmonary carcinoid, occurring in less than 1% at diagnosis and almost 5% during the subsequent course especially in ACs with liver metastases and high tumor burden [14, 30, 31]. Cushing syndrome represents the most frequent paraneoplastic endocrine manifestation in patients with bronchopulmonary carcinoid tumors affecting 4% of patients. A case of elevated parathyroid hormone has been described in a patient with metastatic lung carcinoid [29].

Even if their commonly have an indolent behavior, lung carcinoids are able to metastasize mainly involving regional lymph nodes, liver, bone, adrenal glandes and brain. Therefore, an accurate disease staging based on 8^th^ edition of TNM classification is crucial to choose the best treatment strategy [32]. Disease extension and histologic subtype represent two essential features in the management of patients with bronchopulmonary carcinoids.

The backbone for localized well differentiated lung NENs remains surgery. Systematic hilar and mediastinal lymph nodes dissection is recommended, due to the high risk of metastasis higher in ACs [32]. Complete surgical resection in TCs is associated with almost 90% and 80% survival rates respectively of 5-years and 10-years, and a recurrence rate of 3–5%. Due to their more aggressive course, 5- and 10-years survival rates of ACs are lower (70% and 50% respectively) with a slightly higher recurrence rate (25%) [33–35]. To date, no adjuvant therapy is recommended despite high recurrence rate especially for lung carcinoids with high risk characteristics (atypical histology, high dimensions, lymph nodes involvement), thus clinical trials are needed in this setting [32, 36].

Systemic therapy represents the main option in advanced and unresectable disease (up to 3% of patients are diagnosed with synchronous metastases); accepted choices are somatostatin analogs (SSAs), chemotherapy, everolimus and peptide receptor radionuclide therapy (PRRT). Surgery might be still indicated for symptoms relief; even liver metastasectomy are encouraged in selected cases [37]. Other options for liver lesions are hepatic directed procedures such as ablative therapy, transarterial embolization, transarterial chemoembolization and selective internal radiation therapy [38].

Due to their rarity, there are few prospective studies exclusively regarding lung carcinoids and, to date, only limited therapeutic options are available; thus, novel drugs are strongly needed. In this paper, we extensively reviewed scientific literature firstly exploring available therapeutic options, focusing on molecular basis, mechanisms of resistance and trials ongoing. In the second part, we analyzed new molecular targets and future perspectives in the management of well differentiated neoplasms originated in bronchopulmonary tree.

## Available therapeutic options in lung carcinoids: molecular basis, mechanisms of resistance and trials on going

### SSAs

SSAs represent a classic therapeutic option in the management of lung carcinoids as deduced by almost all specific guidelines [39–41].

Five somatostatin receptors subtypes have been identified (SSTR1, 2A and 2B, 3, 4 and 5) showing a characteristic and distinct distribution among normal cells and tissues; furthermore, a considerable range of solid tumors (breast cancer, lymphoma, hepatocellular carcinoma, renal cell carcinoma, gastric carcinoma and tumors of the nervous system) might express a variable density of SSTRs. However, these receptors represent a distinctive marker only in NENs showing the highest density of SSTRs [42, 43]. Among all SSTRs, subtype 2A (SSTR2A) appears to be the most frequently expressed in lung and gastroenteropancreatic (GEP) NENs, with a relatively lower density in insulinomas, representing the best-studied mediator of somatostatin. Moreover, lung carcinoids show an additional expression of SSTR1, 3 and 5, although in a lower density than SSTR2A, suggesting a possible therapeutic implication of this specific immunostaining pattern [42, 44]. Immunohistochemical (IHC) expression of SSTRs in lung carcinoids seems to be inversely proportional to the aggressiveness of the disease with a lower density in poorly differentiated neoplasms and no significant differences between TCs and ACs. These data might suggest that lung carcinoids and poorly differentiated neuroendocrine tumors of the lung (SCLC and LCNEC) represent two separate entities rather than a continuum of diseases ranging from well to poorly differentiated forms [44, 45]. Besides IHC staining, further in vitro techniques can be utilized to detect SSTRs; moreover, octreotide scintigraphy (SRS) and PET with Ga68-radiolabeled-peptides seem to correlate with IHC expression of SSTRs providing a non-invasive evaluation of this therapeutic biomarker [13].

The importance of SSTRs pathway in NENs management is based on the anti-secretive and anti-proliferative role of somatostatin. Actually, this mediator shows the ability to markedly inhibit hormonal (hyper)secretion and interfere with cell proliferation through direct, promoting cell-cycle arrest and apoptosis, and indirect mechanisms, inhibiting tumor angiogenesis and production of growing factors [46].

The short half-life of somatostatin (< 3 min) limited the usefulness of this neuropeptide in the management of NENs leading to the development of two synthetic somatostatin analogs, octreotide and lanreotide, available in a long-acting formulation. Both octreotide and lanreotide show a high SSTR2A affinity, the receptor predominantly expressed in NENs acting as the principal mediator of these molecules. SSAs were first used in the treatment of acromegaly and pituitary adenomas through the reduction of growth hormone (GH) and insulin-like growth factor-1 (IGF-1) circulating levels [47, 48].

The role of SSAs in the management of well differentiated NENs was initially based on their anti-secretive function. Decades of retrospective and small prospective studies mainly involving patients with GEP-NENs had clearly demonstrated an inhibitory effect on neuropeptides secretion with symptoms control and improvement of quality of life [49–51]. Therefore, SSAs are recommended as first-line option in functioning NENs originating from all sites [39–41]. Furthermore, growing evidences on anti-tumor activity of SSAs in NENs [52–55] have supported the use of these agents also in non-functioning tumors on the basis of two prospective phase 3 randomized trials. In PROMID study, 85 patients with well differentiated (> 95% of patients had Ki-67 values up to 2%), metastatic, functionally active or inactive midgut NENs were randomly assigned to receive either placebo or long acting release (LAR) formulation of octreotide (30 mg every 28 days). Octreotide LAR significantly prolonged time to tumor progression (TTP) (median TTP 14.3 vs 6.0 months; hazard ratio [HR]: 0.34; *p* = 0.000072) and improved stable disease rate (67% vs 37% of patients) regardless of tumor type [56]. Nevertheless, the long-term follow up study showed a similar overall survival (OS) in patients receiving octreotide LAR or placebo (median OS 84.7 vs 83.7 months respectively; HR = 0.83; *p* = 0.51) perhaps due to the effects of crossover [57]. Similarly, in CLARINET trial, lanreotide (120 mg every 28 days) was compared to placebo in 204 patients with advanced, non-functioning, well or moderately differentiated NENs with a Ki-67 index up to 10% originated in pancreas, midgut or hindgut or with unknown origin. Lanreotide significantly improved progression free survival (PFS) (median PFS not reached [NR] vs 18.0 months; HR: 0.47; *p* < 0.001) and HR favored SSA in almost all predefined subgroups with the exception for patients with hindgut tumors representing a small sample [58]. The CLARINET open label extension (CLARINET OLE) study confirmed a consistent PFS (30.8 months) in patients that continued or switched to lanreotide from the core study [59]. Despite the lack of specific prospective trials on octreotide and lanreotide in patients with lung carcinoids, the knowledge acquired from studies in GEP-NENs created the rationale for use of SSAs in non-functioning, well differentiated NENs of lung and other sites. To confirm this, a recent retrospective study evaluated antitumor activity of SSAs in patients with lung carcinoids treated at a referral Centre, showing a clinically meaningful improvement in PFS in the range of the anti-proliferative benefit reported in prospective randomized trials and particularly remarkable in functioning tumors and slowly progressive diseases [60].

To date, two multicentre studies are ongoing to confirm these findings in a prospective way. The SPINET trial (NCT02683941) is a phase 3, randomized, double-blind study evaluating efficacy and safety of lanreotide vs placebo in patients with advanced TCs and ACs, whereas ATLANT (NCT02698410) is a phase 2, multicentre, single arm, open-label trial with the purpose to assess efficacy and safety of the combination of lanreotide with temozolomide (250 mg/day for 5 consecutive days every 28 days for a maximum of 48 weeks) in thoracic well differentiated NENs [61] (Table 1).

**Table 1:** Ongoing clinical trials evaluating the available systemic treatments in lung carcinoids

Systemic treatment	Study identifier	Study title	Study design	Population	Intervention	Primary endpoint
SSAs	NCT02683941	SPINET	Phase 3 Randomized double-blind	TCs and ACs	Lanreotide + BSC vs PCB + BSC	DCR
	NCT02698410	ATLANT	Phase 2 Single arm Open-label	TCs and ACs	Lanreotide + TMZ	PFS
	NCT02823691	MetNET-2	Early Phase 1	Advanced GI-NENs and lung carcinoids	Lanreotide + metformin	Safety
PRRT	NCT03466216	–	Phase 1	SSTRs positive advanced NENs	AlphaMedix™	Safety and DLT
	NCT03273712	–	Phase 2 Single arm Open-label	SSTRs positive advanced NENs	[Yttrium-90-DOTA]TOC	ORR and safety
	NCT03454763	LUTHREE	Phase 2 Randomized Open-label	SSTRs positive advanced NENs	[Lutetium-177-DOTA]TATE every 5 week (intensive) vs Lutetium-177-DOTA]TATE every 8–10 week (no intensive)	PFS and safety
	NCT02754297	P-PRRT	Phase 2 Single arm Open-label	SSTRs positive advanced NENs	[Lutetium-177-DOTA]TOC	ORR
Everolimus	NCT03629847	–	Phase 1–2 Single arm Open-label	SSTRs positive advanced NENs	Everolimus + [Lutetium-177-DOTA]TATE	Safety
	NCT03670030	–	Phase 2 Single arm Open-label	Advanced well differentiated NENs	ABI-009	ORR
CHT	NCT02698410	ATLANT	Phase 2 Single arm Open-label	TCs and ACs	Lanreotide + temozolomide	DCR
	NCT03217097	MGMT-NET	Phase 4 Randomized Open-label	Advanced well differentiated lung, duodenopancreatic and unknown origin NENs	Oxaliplatin vs temozolomide	ORR

*TCs* typical carcinoids, *ACs* atypical carcinoids, *PCB* placebo, *BSC* best supportive care, *TMZ* temozolomide, *DCR* disease control rate, *PFS* progression free survival, *GI* gastrointestinal, *SSTRs* somatostatin receptors, *ORR* objective response rate, *CHT* chemotherapy

The broad and heterogeneous distribution of SSTRs subtypes in normal tissues and solid tumors led to the development of second generation SSAs. These multireceptor-targeted compounds, including SOM230 (pasireotide) and KE108, show the ability to bind to several SSTRs subtypes with high affinity [62, 63]. Targeting multiple SSTRs might be effective in patients with acromegaly or Cushing’s disease become resistant to first generation SSAs (octreotide or lanreotide) as well as in functioning NENs inadequately controlled by octreotide LAR [64, 65]. Given these premises, and considering the additional expression other SSTRs than SSTR2A in lung carcinoids, pasireotide has been evaluated in LUNA trial, an open-label, multicentre, randomized phase 2 study comparing SOM230 vs everolimus vs the combination of pasireotide with everolimus in advanced thoracic (lung and thymic) carcinoids. Pasireotide alone or in combination with everolimus showed preliminary evidence of activity with an acceptable safety profile; however, the study met the primary end point in all three arms failing to demonstrate a superior efficacy, therefore further studies are needed [66]. Despite this limitation, LUNA represents the first and only prospective, randomized study exclusively dedicated to patients with advanced thoracic well differentiated NENs, strengthening the concept of realize clinical trials in homogeneous population to get a perfect tailoring of available medical therapies according to precision medicine.

The emerging of resistance during treatment frequently limits the clinical utility of SSAs. Resistance to SSAs has been primarily attributed to a primary low or an acquired reduction in SSTR2 expression, whilst a higher SSTR2-to-SSTR5 ratio correlates with biochemical control under octreotide treatment [43]. On this basis, SSAs are particularly recommended as first line therapy in advanced lung NENs with highly positive SRS/PET with 68 Ga-DOTA-peptide and low proliferative index (Ki-67 < 10%) [39]. Other rare mechanisms of resistance include mutation of SSTR2 or SSTR5 and signaling defect [43].

Concomitant administration of SSAs with other molecules might represent a promising strategy to avoid or delay the development of drug resistance. Among targeted agents, there is a strong rationale for combining SSAs with everolimus: mTOR inhibition induces an upregulation of upstream signaling primarily mediated by IGF-1 pathway, while SSAs seem to reduce serum concentration of IGF-1 potentially reversing this feedback loop [67, 68]. The promising activity of this combination has been assessed in an open-label phase 2 trial evaluating the activity of everolimus either 5 mg/day or 10 mg/day in combination with octreotide LAR in 60 patients with advanced low to intermediate grade NENs (lung carcinoids in 4 patients). The overall response rate (ORR) reached 20%, with a median PFS of 60 weeks [69]. These results were confirmed in 160 patients with well to moderately differentiated, advanced pancreatic NETs progressed after cytotoxic chemotherapy and stratified by prior octreotide therapy, enrolled in a similar multicentre, open-label phase 2 trial (RADIANT 1) [70].

Preliminary evidences regarding SSAs seem to suggest an inhibitory effect on tumor angiogenesis suppressing the release of angiogenic factors such as vascular endothelial growth factor (VEGF) [71]. On the basis of these data, the combination of SSAs with metronomic chemotherapy and anti-angiogenetic agents might produce a synergistic effect, as confirmed in a pilot study evaluating the association of metronomic temozolomide (100 mg/day continuously), bevacizumab and octreotide LAR in 15 patients with advanced NETs progressed on previous therapies (1 patient with lung carcinoid) assessing a median TTP of 36 months [72]. Other promising combinations in lung carcinoids might include dopamine agonists (DAs) mainly directed against dopamine receptor 2 (DR2), that seems to be overexpressed in thoracic well differentiated NENs [44], and metformin through the reduction of IGF-1 circulating levels and the inhibition of mTOR pathway [73]. A pilot, open-label, prospective study is ongoing to evaluate safety of lanreotide in combination with metformin in patients with advanced, well differentiated gastrointestinal or lung carcinoids (NCT02823691) [61] (Table 1).

### PRRT

PRRT represents a kind of systemic radiotherapy based on intravenous administration of radiolabeled SSAs. The radiopeptides consist of a radionuclide bound to a carrier molecule through a chelator, such as DOTA (tetraazacyclododecane-tetra-acetic acid) or DTPA (diethylenetriamine penta-acetic acid). Due to their peculiar conjugation, radiolabeled SSAs are able to bind SSTRs localized on the surface of neuroendocrine cells. Upon receptor targeting, radiopeptides are internalized into neoplastic cells delivering their own radioactivity into the intracellular compartment of the neoplasm [74]. On this basis, key criteria in patient selection for PRRT are overexpression of SSTRs as well as the evidence of their functionality. SRS and PET with Ga68-radiolabeled-peptides represent the most rigorous non-invasive method to identify an overexpression of functioning SSTRs in vivo and simultaneously in all detectable lesions. False positives (gallbladder, accessory spleens, recent surgery, radiotherapy and all conditions associated with granulomatous-lymphoid infiltrates) and false negatives (small lesions, insulinomas) represent potential limitations of functional imaging techniques. Immunohistochemistry and the more accurate molecular analyses (polymerase chain reaction—PCR and Western blot—WB) might be used as an alternative method though limited to the single examined lesion [75, 76].

The first isotope to be experienced was Indium-111 characterized by the emission of Auger and conversion electrons with a medium-to-short tissue penetration. Indium-111 achieved not really satisfactory results with a low proportion of partial response maybe due to suboptimal treatment of large tumors [77]. Due to these partially disappointing results, new isotopes with higher energy and longer range have been evaluated such as the pure β-emitter Yttrium-90 and the β-γ-emitter Lutetium-177 resulting in a greater therapeutic potential with acceptable tolerability [74].

PRRT consists of systemic infusion of these suitably radiolabeled synthetic SSAs. The fractionated administration of radiopeptides in sequential cycles every 6 to 9 weeks seems to be necessary to recover from hematological toxicity. The cumulative dose mainly depends on the irradiation of kidneys and bone marrow, which represent dose-limiting organs. Positively charged amino acids (lysine or arginine) are usually coadministrated to decrease renal irradiation causing possible gastrointestinal symptoms (nausea, emesis) [78].

PRRT has given a significant contribution in NENs management concurring to the remarkable expansion of therapeutic landscape. Although lungs represent the second most common primary site, data regarding the role of PRRT in TCs and ACs mainly result from general studies on NENs including a minor percentage of lung carcinoids. Imnof et al. realized one of the largest studies evaluating response, survival and safety profile of an Yttrium-90 labelled radiopeptide in 1109 patients with 25 different neuroendocrine cancer subtypes including 84 lung carcinoids. In this phase II single-center open-label trial, [Yttrium-90-DOTA]-TOC achieved morphologic response in 378 patients with an ORR of 34.1%; in the subgroup of lung carcinoids ORR was 28.6% with no complete responses. Median survival was 2.9 times longer than the expected value detected for G1-G2 advanced NENs [24] with a longer survival correlated to high tumor baseline uptake and morphological, biochemical and clinical response. The most relevant adverse events were transient hematological (grade 3 and 4 in 12.8% of patients) and permanent renal toxicities (grade 4 and 5 in 9.2%) [79]. A more recent study enrolled a large population of 610 Dutch patients with bronchial (23 patients) or GEP-NENs treated with ≥ 100 mCi (3.7 GBq) [Lutetium-177-DOTA]-TATE evaluating long-term efficacy, survival and tolerability. In lung carcinoid cohort, PRRT obtained 30% ORR, again without complete responses, and an additional 30% of stable disease. PFS and OS for lung carcinoids were 20 and 52 months, respectively, with lower values than global enrolled population (global PFS 29 months and global OS 63 months) [80].

Two recent studies have evaluated the role of PRRT in a homogeneous population of lung carcinoids. In the first study, 114 patients with advanced bronchopulmonary carcinoid treated in a referral Centre with three different PRRT protocols ([Yttrium-90-DOTA]TOC vs [Lutetium-177-DOTA]TATE vs [Yttrium-90-DOTA]TOC + [Lutetium-177-DOTA]TATE) were retrospectively evaluated. The estimated median OS and PFS were 58.8 and 28.0 months, respectively. Independent factors significantly associated with both death and disease progression were age at PRRT and previous chemotherapy; furthermore, longer OS and PFS were achieved in cases of objective response, with a global ORR of 26.5%. The cohort of 21 patients treated with a combination of both radiopeptides obtained the highest ORR and PFS rates at 3 years after the start of PRRT (38.1% and 46.2%, respectively), while treatment with [Lutetium-177-DOTA]TATE was associated with the highest 5-year OS (61.4%) and the lowest rate of adverse events [81]. In the second study, 34 consecutive patients with progressive advanced lung carcinoids treated with four or five cycles of [Lutetium-177-DOTA]TATE have been prospectively evaluated. PRRT achieved 15% ORR in the entire population with 3% of complete response (all TCs), while median PFS and OS were 18.5 and 48.6 months, respectively. Regarding to histological type, TCs obtained a higher percentage of disease control rate (DCR) (80% vs 47%) and better survival in terms of PFS (20.1 vs 15.7 months) as well as OS (48.6 vs 37.0 months). No major acute or delayed adverse events were observed globally, partially due to amino acid (lysine) administration that optimize renal protection [82].

In this trial, Ianniello et al. also evaluated the prognostic role of PET with fludeoxyglucose (FDG) status and thyroid transcription factor 1 (TTF-1) immunostaining in advanced bronchopulmonary carcinoids treated with PRRT. FDG PET-positive patients showed worse survival (in terms of OS and PFS) recognizing FDG PET-positivity, found more frequently in ACs than TCs, as a new hallmark of disease aggressiveness. TTF-1 expression seems to resemble FDG PET-positivity with higher percentage of positivity in ACs than TCs (79% than 20%) and lower survival. Therefore, patients who showed TTF-1 and FGD-PET negativity seem to get the best outcome after PRRT while positivity of TTF-1 and/or FDG PET might suggest choosing more aggressive treatment options [82]. However, the most important predictive biomarker still remain SSTRs expression: higher ORR have been observed in NENs showing grade 4 uptake by Krenning score (in case of SRS) or a maximum standardized uptake value (SUV) > 16 in PET with Ga68-radiolabeled-peptides) [83, 84].

Accordingly to all these data, PRRT might represent an effective option to treat patients with advanced TCs and ACs demonstrating an overexpression of functioning SSTRs [39]. However, the burning debate regarding the best timing to use PRRT still remains open: the lack of solid evidences deriving from large, prospective, advanced-phase studies evaluating pulmonary carcinoids would suggest to choose primarily therapies supported by higher level of evidence. At the same time, reserving PRRT to more advanced stages of the disease could led to PRRT being less effective due to biological events (SSTRs reduction, development of further genetic mutations such as TP53) that would make the disease more resistant to this treatment regardless of the histological subtype [13, 85, 86]. Furthermore, some data seem to suggest that previous chemotherapy might negatively influence PRRT safety and efficacy [81]. Randomized trials are needed to better define the optimal therapeutic sequence and identify which radiopeptide, alone or in combination, is the most effective and safest in patients with lung carcinoids.

To date, NETTER-1 represents the only phase 3 multicentre randomized trial evaluating efficacy and safety of [Lutetium-177-DOTA] TATE in small intestinal NENs. In this study, 229 patients with advanced, progressive, well differentiated midgut NENs were randomly assigned to receive either four cycles of PRRT or high dose of octreotide LAR (60 mg every 4 weeks). Treatment with [Lutetium-177-DOTA]TATE significantly prolonged PFS (NR vs 8.4 months; HR: 0.21; *p* < 0.001) and improved ORR (18% vs 3%); in the planned interim analysis of OS, risk of death was 60% lower in experimental arm (HR: 0.40; *p* = 0.004). No renal toxicities have been observed in the enrolled population [87]. Two phase 2 trials are ongoing with the purpose to evaluate efficacy and safety of PRRT with Yttrium-90 (NCT03273712) or Lutetium-177 (NCT02754297) in NENs of various origin including lung carcinoids [61] (Table 1).

Combination of PRRT with chemotherapeutic agents have been tested in early studies performed in patients with well differentiated NENs including low cases of lung carcinoids. Antimetabolites (capecitabine or 5-fluorouracil [5-FU]) and/or temozolomide in combination with Lutetium-based PRRT achieved the best results in terms of ORR [88, 89]. Furthermore, combined therapies with other innovative agents (everolimus, PARP-inhibitors, etc.) have preliminarily shown promising results [90, 91]. To date, no studies have compared PRRT in combination with other treatments versus the same agents used in a sequential strategy: the higher value of ORR achieved with a combinational approach should not be considered as a detector of better survival, especially in slowly progressive neoplasms with limited therapeutic options. The high rate of tumor shrinkage might lead to consider PRRT, alone or in combination with chemotherapy, a very promising approach in the neoadjuvant setting, as shown in several case series especially regarding locally advanced or oligometastatic pancreatic NENs [92–94]. The downstaging achieved by PRRT might be helpful also in bronchopulmonary carcinoids making the surgical intervention possible and effective, as seen with chemotherapy for the more common adenocarcinomas and squamous cell carcinomas of the lung [95].

Although both effective, Yttrium-90 and Lutetium-177 show a dissimilar penetration range, targeting large and small lesion respectively, and a slightly different safety profile with a minor risk of severe nephrotoxicity with Lutetium-177 [74]. Considering the differences among the available radioisotopes, a combined treatment with alternate administrations of Yttrium-90 and Lutetium-177 might attain a better outcome as shown in several non-randomized trials [96, 97]. These results were confirmed in a large cohort study comparing repeated injection of [Yttrium-90-DOTA]TOC with alternate cycles of [Yttrium-90-DOTA]TOC and [Lutetium-177-DOTA]TOC in 486 patients with advanced NENs of various origin. Combined therapy achieved a significantly longer survival than monotherapy with [Yttrium-90-DOTA]TOC (5.51 vs 3.96 years; HR: 0.64; *p* = 0.006) with a comparable rate of severe toxicities in both groups [98]. Furthermore, new schedules might improve risk–benefit ratio of PRRT. A randomized phase 2 trial is ongoing evaluating efficacy and safety as co-primary objective of two different schedule (intensive vs no intensive) of [Lutetium-177-DOTA]TATE administrations in SSTRs positive NENs (NCT03454763) [61] (Table 1).

In recent years, there has been growing interest about new generation compounds as α-emitting radioisotopes, characterized by higher energy deposition in tissue with a shorter path length than β-particles. The high potency together with an exquisite specificity allow α-emitting radioisotopes to sterilize individual cancer cells sparing healthy tissue. The α-emitting radiopeptides [Actinium-225-DOTA]TOC and [Bismuth-213-DOTA]TOC have shown a promising antitumor activity with an acceptable safety profile in animal studies as well as in a pilot study of 7 patients with advanced NENs progressing after standard PRRT [99–101]. A phase 1 trial (NCT03466216) is ongoing to evaluate safety and dose limiting toxicity (DLT) using ascending doses of AlphaMedix™, an α-emitter (Plumbum-212) radiopeptide, in adult patients with SSTRs positive NENs [61] (Table 1).

### Chemotherapy

Well differentiated tumors are generally less responsive to chemotherapy than poorly differentiated neoplasms because of their slowly progressive nature. Only few studies have described the role of systemic chemotherapy in lung carcinoids [102–104]. The drugs used in this context include 5-FU, capecitabine, doxorubicin, dacarbazine, streptozocin, cyclophosphamide, platinum derivatives, etoposide and temozolomide, but all have shown limited efficacy (< 30%) [32].

Temozolomide, an oral agent with a low toxicity profile, represents an interesting drug tested in few studies. The first trial evaluating this drug in bronchopulmonary carcinoids was a retrospective study: chemotherapy was tested in a mixed population of NENs, including 13 patients with lung well differentiated NENs (10 TCs and 3 ACs) in second or subsequent lines of therapy. Eight patients exhibited clinical benefit and 3 of them were TCs [105]. Similar results were found in a bigger retrospective cohort study including 31 metastatic lung carcinoids: 3 partial responses (PR) were achieved in patients with ACs and high Ki-67 index [104]. Temozolomide could represent an interesting choice even in case of brain metastasis [106]. Methylguanine DNA methyltransferase (MGMT) (the enzyme responsible for guanine methylation that endorses DNA repairing) expression in tumor tissue might help to screen responders: protein deficiency determines response to temozolomide in pancreatic NENs [107]. However, to date this test needs more validation before being used in clinical practice to influence chemotherapy choice.

An interesting Italian phase 2 clinical trial (ATLANT) evaluating the combination of temozolomide and lanreotide in patients with unresectable, advanced lung and thymic well differentiated NENs is ongoing. This trial will also investigate the role of MGMT on tumor tissue and methylation of its promoter in blood (NCT02698410) [61] (Table 1). The role of MGMT methylation as predictive factor of the response to alkylating agents represents the primary aim of a phase IV trial evaluating patients with a duodenopancreatic or lung carcinoids or well differentiated NENs of unknown origin treated with chemotherapy with alkylating agents or oxaliplatin (NCT03217097) [61] (Table 1).

Moreover, a phase 2 trial has described the synergic use of capecitabine and temozolomide (CAPTEM) against several NENs included lung carcinoids: patients with well to moderately differentiated metastatic diseases (Ki-67 ≤ 20%) achieved one CR, four PR and seven stable disease (SD) [108]. Another combination explored in lung carcinoids was temozolomide + thalidomide achieving a 7% of ORR [109, 110].

The doublet etoposide plus platinum derivatives represents the regimen of choice in poorly differentiated NENs, but it was tested even in small series of lung carcinoids, as seen in two retrospective study: Forde et al. enrolled 17 patients with advanced bronchopulmonary carcinoid achieving an ORR of 23.5%, whilst a CR and 9 (69%) SD (stable disease) have been achieved in 13 patients with ACs treated with platinum derivatives and etoposide [102, 111].

About platinum combined therapies, Turner et al. reported the outcome of patients with locally advanced or metastatic NENs (lung carcinoids was reported in 8 patients) treated with 5-FU, cisplatin and streptozocin. Treatment with triplet was associated with an ORR of 33% (25% for non pancreatic primary sites), while SD occurred in 51%, with progression in 16%; regimen was well tolerated [112].

In 2006, Italian researchers evaluated the efficacy of oxaliplatin and capecitabine (XELOX) in 40 patients with advanced NENs including 13 untreated poorly differentiated tumors and 27 well differentiated NENs progressed after somatostatin analogues; among these 10 patients had lung NETs. Among 27 patients with well differentiated NENs, 8 PR (30%) and 13 SD (48%) were achieved, whilst worse results were shown in poorly differentiated forms, suggesting that XELOX regimen is more effective and well tolerated in well differentiated diseases progressed to somatostatin analogues [113].

Furthermore, Cassier et al. described the association of gemcitabina with oxaliplatin (GEMOX) in most cases administrated in second or subsequent treatment lines in 20 patients (4 with lung NENs) with progressive disease: three (17%) out of 18 patients achieved a PR [114].

Accordingly to these evidences, a standard of care regarding chemotherapy in lung carcinoids is still lacking and current guidelines suggest to use cytotoxic drugs when all others systemic treatments have failed, in case of rapid disease progression and in absence of SSTRs. Platinum derivatives plus etoposide should be used for ACs with high proliferation index, whilst temozolomide represents the treatment of choice in all metastatic lung carcinoids [39–41].

### Everolimus

Everolimus is a selective inhibitor of mTOR (mammalian target of rapamycin) with antitumor activity in patients with advanced NENs.

mTOR represents a key component involved in cell growth, proliferation and survival that has been explored with interest also in lung NENs [115]. A lower expression of active forms of mTOR and protein-kinasi 1 p70-S6 (acting as mTOR target) was detected in carcinomas of either large or small cell types, whereas mTOR pathway results frequently activated in pulmonary carcinoids [116].

Mediators involved in mTOR pathway, downstream and upstream to mTOR complex, might be candidate to work as potential biomarkers of response to mTOR inhibition. Bronchial carcinoid cells of patients responding to mTOR inhibitors were shown to harbor, indeed, higher levels of phosphorylated mTOR [117]. Moreover, additional players might interact with mTOR pathway. Preliminary evidences seem to suggest an inverse correlation between GLUT-1 (glucose transporter 1) expression and mTOR signaling; furthermore, the expression of these molecules seem strongly associated with SSTR2A density, suggesting that a synergistic effect might be obtained combining these treatments as showed in intestinal NENs [13, 118].

To date, two mTOR inhibitors are available as antineoplastic drugs. Temsirolimus is an ester analog of sirolimus (or rapamycin) administered intravenously. Everolimus is a rapamycin derivative with a hydroxyethyl group that makes the compound more water soluble and so orally administrable; [119] it works by preventing phosphorylation of mTOR complex 1 (mTORC1) effectors, such as eukaryotic initiation factor 4E binding protein-1 (4E-BP1) and protein S6 kinase (p70S6 K), two players involved in proteins translation.

The role of everolimus in lung carcinoids has been firstly investigated in RADIANT 2 trial, a randomized, double-blind, placebo-controlled phase 3 study, that assessed the combination of everolimus plus octreotide LAR vs placebo plus octreotide LAR in 429 patients with low or intermediate grade NENs with different origins (included 44 lung carcinoids) and carcinoid syndrome. Combination arm (everolimus plus octreotide LAR) provided a clinically meaningful 5.1 months improvement in PFS (16.4 vs 11.3 months; HR: 0.77; *p* = 0.026) with control of tumor growth and a significant reduction in biochemical markers. Treatment benefit was recorded irrespective of previous chemotherapy or SSAs exposure, WHO performance status, age, gender, tumor grade and tumor primary site. The safety of the experimental arm was acceptable with a low rate of severe adverse events and comparable to the known safety of the singular drugs [118]. Though not statistically significant and affected by potential bias, such as heterogeneity of the enrolled population, imbalances in baselines characteristics and crossover design, this study clearly supported the efficacy of everolimus in advanced NENs.

An interesting analysis was made in the subgroup of NENs with lung primary site (44 patients): 33 received the combined treatment whilst 11 were included in placebo arm. The addition of everolimus to octreotide LAR achieved an increasing in PFS of 2.4 fold (8 months, from 5.59 to 13.36 months) with a reduction of risk of progression equal to 28%. This result, even if not significantly, represented a meaningful improvement suggesting that the addition of everolimus should be considered an option in lung carcinoid, also considered the limited therapeutic chances of this patients [120].

In RAMSETE trial, an open-label, multicentre, phase 2 study, the safety and efficacy of everolimus in monotherapy were evaluated in 73 patients with advanced non pancreatic NENs without carcinoid syndrome. A high rate of disease stabilization (the best response by central review was stable disease in 55% of patients) was achieved after prior tumor progression with favorable PFS value (PFS by central review was 185 days). This study further supports efficacy of everolimus in other types of NENs than those studied in RADIANT-3 (pancreatic NENs) and RADIANT-2 (NENs associated with carcinoid syndrome) [121].

On these basis, another randomized, double bind, phase 3 study has been realized (RADIANT-4) leading to worldwide approval of everolimus for the treatment of patients with advanced, progressive, well differentiated, non-functioning, gastrointestinal and lung NENs. This trial enrolled 302 patients (included 90 lung carcinoids) randomly assigned to receive everolimus (n = 205) or placebo (n = 97). Everolimus achieved an improvement in PFS of 7 months (11.0 vs 3.9 months; HR: 0.48; *p* < 0.00001) and a 52% reduction in the risk of disease progression or death. The first interim analysis of OS suggested a trend toward improved survival in favor of everolimus, although this result was not statistically significant. Treatment toxicity was almost always manageable [122].

In the subcohort of 90 lung NENs, the largest series of lung carcinoids ever included in a phase 3 trial, the 63 patients who received everolimus had a clinical improvement in PFS (5.6 months) compared to placebo. Therefore, the findings of RADIANT-4 have validated the role of mTOR pathway in NENs of lung or gastrointestinal origin [123].

Based on RADIANT-4 results and supported by RAMSETE and RADIANT-2 trials, everolimus might be considered a valid treatment option in advanced well differentiated progressive lung NENs [39–41]. LUNA trial, the only prospective, randomized study exclusively regarding patients with advanced thoracic well differentiated NENs comparing pasireotide vs everolimus vs the combination of pasireotide, has given another significant support to everolimus efficacy in bronchopulmonary carcinoids (see SSAs paragraph) [66].

To date, a univocal treatment strategy in lung carcinoids has not been identified yet, thus everolimus might be recommended as a first or second line therapy in progressive and metastatic disease [41]. A retrospective analysis evaluated the role of everolimus related to previous treatment in 169 patients with advanced progressive NENs, included 84 non pancreatic tumors. This study confirmed the importance of everolimus in these neoplasms achieving similar efficacy rates and safety profile regardless of the primary site. One of the most interesting data detected is a significant increase of everolimus’ severe adverse events in patients long time pretreated with chemotherapy and PRRT (grade 3–4 reported in 86.8% vs 34.3% respectively). Therefore, searching for the best therapeutic algorithm, therapy with everolimus should be planned before PRRT and chemotherapy to avoid the onset of predictable severe toxicities that might limit subsequent treatments [124].

The mTOR pathway is a complex system of factors, thus understanding the mechanisms underlying resistance to everolimus might prevent and delay disease progression and treatment discontinuation. Everolimus specifically inhibits mTORC1 and consequently the cascade of p70S6 K/4E-BP1 decreasing protein synthesis and cell growth [125–130]. The PI3K (phosphatidylinositol 3-kinases)/Akt (Protein kinase B)/mTOR pathway might be activated also by upstream factors such as IGF-1, leading to mTOR-mediated phosphorylation and degradation of insulin receptor substrate-1 (IRS-1), the man mediator of IGF-1 [131–134]. mTORC1 inhibition might led to suppression of this balancing feedback, causes an over-activation of upstream signaling, including PI3K/Akt [135, 136]. Furthermore, the inability of everolimus to block mTORC2 might induce upstream Akt phosphorylation [125, 137, 138]. Mitogen activated protein kinase (MAPK) activation, up-regulation of pro-angiogenic factors and activation of Ras pathway might represent other potential mechanisms of resistance to mTOR inhibitors [135, 139–141].

Dual inhibition of PI3K/Akt and mTOR pathway could represent a therapeutic approach able to overcome everolimus resistance [137, 142–144]. On this basis, a phase 2 trial randomly compared efficacy and safety of BEZ235, a dual PI3K/mTOR inhibitor, and everolimus in 62 patients with advanced NENs. Unfortunately, BEZ235 did not demonstrated superior efficacy and treatment was affected by high toxicity leading patients to require frequent dose modifications and treatment discontinuations. As a result, the shorter treatment duration of BEZ235 compared to everolimus (22.9 vs 39.4 weeks) had negative impact on its anti-tumor activity. Further studies using other agents with better tolerability are warranted to clarify the effect of pan-PI3K inhibitors against resistance to everolimus [145].

Synergistic effect of everolimus combined with SSAs seem to result from the action of the these drugs on both tumor cells and their microenvironment through a common molecular target, the PI3K/AKT/mTOR signaling pathway [146, 147]. Their synergistic effect was investigated in RADIANT 2 [118]. Furthermore, a phase 2 multicentre trial assessed the efficacy and safety of first-line therapy with everolimus plus octreotide LAR in advanced mixed NENs with or without carcinoid syndrome; the combination achieved an ORR of 20% in agreement with previous data on the efficacy of everolimus in NENs [148]. These findings supplement those reported in the RADIANT-2 in terms of improvement of PFS, tolerability and safety profile of these drugs. Furthermore, the LUNA trial suggested an improvement of PFS with combination everolimus plus pasireotide LAR in patients with advanced thoracic carcinoid [66].

The VEGF signaling pathway acts through the PI3K/mTOR pathway and the PI3K pathway is critical for endothelial cell activation and tumor angiogenesis. Thus, combining antiangiogenic compounds with mTOR inhibition might maximize inhibition of tumor angiogenesis [149]. The co-administration of bevacizumab with targeted agents has been studied in several phase 2 trials, including CALBG 80701, that enrolled 150 patients with pancreatic or gastrointestinal carcinoids (no data exist in lung carcinoids). In this randomized phase 2 study the combination of bevacizumab plus everolimus resulted in increased ORR and a greater but not statistically significant PFS, but was accompanied with higher toxicity compared to everolimus monotherapy [150]. This therapeutic approach deserves investigation in advanced bronchopulmonary carcinoids. The synergy between VEGFR pathway and mTOR inhibitor had also inspired a phase I study to evaluate the safety and feasibility of combing sorafenib and everolimus in patients with advanced NENs (included 3 bronchial carcinoids). Unfortunately, this combination comported unacceptable toxicities that limited the escalation to the anticipated full doses of both agents administrated together [151].

Based on a potential synergistic effect, several trials evaluating combined treatment with everolimus (or temsirolimus) in patients with advanced NENs are ongoing.

Regarding lung carcinoids, a phase 1–2 study is ongoing aiming to assess safety and efficacy of everolimus combined with intravenous radiolabeled [Lutetium-177-DOTA]TATE as first line therapy in unresectable well to moderately differentiated NENs of gastrointestinal, lung or pancreatic origins (NCT03629847) [61] (Table 1). Another interesting phase 2 study will try to determine the efficacy of ABI-009, a human albumin-bound rapamycin intravenously administrated, in advanced well differentiated NENs of lung, gastrointestinal tract or pancreas origin progressed or intolerant to everolimus (NCT03670030) [61] (Table 1).

## New molecular targets and future therapeutics perspectives

### Angiogenesis

Angiogenesis represents neovascularization of tumors and VEGF-A is the main mediator binding to its tyrosine-kinase receptor family. The role of angiogenesis as a challenging therapeutic target has led to the development of specific inhibitors of both VEGF and its receptors [152]. The endocrine phenotype of NENs architecture together with the ability to synthetize high levels of VEGF-A justify the hypervascularization of these neoplasms that represents one of their main distinctive markers [153]. Accordingly to these findings, targeted antiangiogenic agents have been successfully explored in GEP-NENs leading to approval of sunitinib in advanced well differentiated pancreatic NENs [154].

Angiogenic factors as single nucleotide polymorphisms (SNPs) of VEGFs and its receptors might play a prognostic and predictive role of in lung NENS as shown in the retrospective study conducted in GEP-NENs patients [155].

The anti-VEGF monoclonal antibody bevacizumab represents the first antiangiogenic drug tested in a mixed population of NENs including four patients with lung carcinoids. In this randomized phase 2 trial, bevacizumab achieved favorable results in terms of ORR, reduction of blood flow and longer PFS compared to pegylated interferon (PEG-IFN) [156]. Based on these results, a subsequent phase 3 trial was realized to access PFS of bevacizumab against interferon alfa-2b (IFN-α-2b) both added to octreotide LAR in patients with advanced NENs. Unfortunately, although a longer TTP in favor of the monoclonal antibody maybe due to high number of withdrawn in control arm, no significant differences in PFS were observed between arms [157].

Sunitinib malate is a small molecule working as multitargeted receptor tyrosine-kinase inhibitor (TKI) with antiangiogenic activity, established as a standard of care in renal cell carcinoma, GIST (gastrointestinal stromal tumor) and pancreatic NENs. Data regarding the role of sunitinib in lung carcinoids derive solely from a phase 2 trial evaluating two different cohorts. Among extra-pancreatic NENs, sunitinib achieved a rather low ORR (2.4% whilst 16.7% in pancreatic NENs) with an interesting PFS of 10.2 months (7.7 months in pancreatic NENs) and one-year survival rate reaching 84% [158]. The mainly cytostatic effect of sunitinib in indolent diseases as extra-pancreatic well differentiated NENs should be further investigated in randomized trials.

In the phase 2 PAZONET study, Grande et al. evaluated activity of pazopanib, another multitargeted antiangiogenic TKI, in 44 patients with advanced NENs (including 5 bronchopulmonary carcinoids) after failure of systemic therapies. Pazopanib achieved a PFS of 9.5 months, whilst the 6-month CBR (clinical benefit rate) varied according to previous treatment with a global value of 59.5%; however, results were quite disappointing in the subgroup of thoracic (lung and thymic) NENs (PFS = 3.4 months) [159]. Combination with octreotide LAR does not seem to improve responses in extra pancreatic carcinoids, as shown in another phase 2 trial evaluating pazopanib in 52 advanced NENs [160]. A randomized phase II trial is ongoing assessing efficacy (PFS) of pazopanib versus placebo in patients with advanced pretreated carcinoid tumors (NCT01841736) [61] (Table 2).

**Table 2:** Ongoing clinical trials evaluating potential therapies in lung carcinoids

Molecular target	Study identifier	Study title	Study design	Population	Intervention	Primary endpoint
Angiogenesis	NCT01841736	–	Phase 2 RANDOMIZEDDOUBLE-blind	Advanced well differentiated NENs	Pazopanib vs PCB	PFS
	NCT00605566	–	Phase 2 Single arm Open-label	Advanced well differentiated NENs	Sorafenib + metronomic cyclophosphamide	ORR
	NCT02795858	–	Phase 2 Single arm Open-label	Advanced extra-pancreatic well differentiated NENs	Ramucirumab + SSA	PFS
	NCT01782443	–	Phase 2 Single arm Open-label	Advanced well differentiated NENs	Ziv-aflibercept	PFS
	NCT03375320	CABINET	Phase 3 Randomized double-blind	Advanced well differentiated NENs	Cabozantinib vs PCB	PFS
	NCT02588170	–	Phase 3 Randomized double-blind	Advanced extra-pancreatic well differentiated NENs	Surufatinib vs PCB	PFS
	NCT02259725	–	Phase 2 Single arm Open-label	Advanced well differentiated NENs	Regorafenib	PFS
	NCT01744249	–	Phase 2–3 Randomized double-blind	Advanced extra-pancreatic well differentiated NENs	Axitinib + Octreotide LAR vs PCB + Octreotide LAR	PFS
	NCT02399215	–	Phase 2 Single arm Open-label	Advanced extra-pancreatic well differentiated NENs	Nintedanib	PFS
ErbB	NCT00843531	–	Phase 2	Moderately to well differentiated advanced NENs	Everolimus + Erlotinib	ORR
ALK	NCT02568267	STARTRK-2	Phase 2 Basket study	Solid tumors (including NENs) harboring NTRK 1/2/3, ROS1 or ALK rearrangement	Entrectinib	ORR
CDK 4/6	NCT02420691	–	Phase 2 Single arm Open-label	Foregut advanced well differentiated NENs	Ribociclib	ORR
	NCT03070301	–	Phase 2 Single arm Open-label	Foregut advanced well differentiated NENs	Ribociclib + Everolimus	PFS
Immuno-therapy	NCT02955069	–	Phase 2 Single arm Open-label	Advanced well differentiated NENs of pancreatic, GI or thoracic origin and advanced GEP-NECs	PDR-001	ORR
	NCT03278379	NET-002	Phase 2 Single arm Open-label	Advanced well differentiated NENs	Avelumab	ORR
	NCT03420521	–	Phase 2 Single arm Open-label	Advanced well differentiated NENs of pancreatic, GI or lung origin (3 cohorts)	Nivolumab + Ipilimumab	ORR
	NCT02923934	–	Phase 2 Single arm Open-label	Rare cancers (including NENs)	Nivolumab + Ipilimumab	CBR
	NCT03095274	DUNE	Phase 2 Single arm Open-label	Advanced well differentiated NENs of pancreatic, GI or lung origin and advanced GEP-NECs (4 cohorts)	Durvalumab + Tremelimumab	CBR
	NCT03728361	–	Phase 2 Single arm Open-label	Progressive SCLC and advanced NENs (2 cohorts)	Nivolumab + Temozolomide	ORR

*PCB* placebo, *PFS* progression free survival, *ORR* objective response rate, *SSAs* somatostatin analogs, *ORR* objective response rate, *GI* gastrointestinal, *GEP-NECs* gastroenteropancreatic neuroendocrine carcinomas, *CBR* clinical benefit rate, *SCLC* small cell lung cancer

Targeted therapies with antiangiogenic properties have been also investigated in combination with chemotherapy. Chan et al. realized a phase 2 study evaluating bevacizumab plus temozolomide in 34 patients with advanced NENs (including 4 lung carcinoids) achieving promising results in pancreatic tumors [110]. The angiogenesis suppression obtained with metronomic chemotherapy might suggest a synergistic effect with antiangiogenic agents, as seen in a pilot study conducted in 15 patients with advanced NENs (1 lung carcinoids) treated with bevacizumab, metronomic temozolomide and octreotide LAR achieving an ORR of 64% with an acceptable safety profile [72]. Another phase 2 trial (NCT00605566) investigated the combination of sorafenib plus metronomic cyclophosphamide in patients with progressive moderately to well differentiated NENs, but no results are available yet [61] (Table 2).

Owing to the growing interest about targeted therapy, many antiangiogenic agents are currently evaluated in prospective trials involving mixed population of advanced NENs including bronchopulmonary carcinoids [61] (Table 2).

### ErbB

ErbB signaling and other related pathways constitute a complex network of interactions crucially involved in cell growth, survival, proliferation and differentiation. To date, four ErbB tyrosine-kinase receptors has been identified leading to several biologically meaningful combinations: ErbB1 or epidermal growth factor receptor (EGFR/ErbB1/Her1), ErbB2 (Her2/neu), ErbB3 (Her3) and ErbB4 (Her4). Overexpression and/or mutation of ErbB receptors are frequently involved in tumorigenesis of NSCLC (non small cell lung cancer), breast and ovary cancer and many other neoplasms providing a “druggable” molecular target [161].

Rickman et al. first investigated the role of ErbB pathway in lung carcinoids analyzing IHC staining for Erb2 receptors and sequencing DNA of EGFR gene (exons 18 to 21) in 31 surgically resected specimens. EGFR expression was detected in 45.8% of TCs and 28.6% of ACs, while almost all carcinoids showed a considerable staining for ErbB3 and ErbB4 as well as a totally lack of ErbB2 expression. Nevertheless, DNA sequencing of EGFR and KRAS revealed the absence of activating mutations [162]. Recent studies analyzed genomic profile of low-grade lung carcinoids sequencing several genes related to therapy response. EGFR expression was observed in a considerable rate of the investigated tumors (87.5% of TCs add 100% of ACs), suggesting a potential role of monoclonal anti-EGFR-antibodies in the management of advanced bronchopulmonary carcinoids [163, 164].

Accordingly to these results, Bendell et al. evaluated safety and activity of HER1/HER2 inhibitor pertuzumab combined with anti-VEGF monoclonal antibody bevacizumab and octreotide LAR in advanced NENs, lung carcinoids included, achieving an ORR of 16% with 2 cases of complete response (CR) and a low incidence of severe toxicities [165]. Another phase 2 trial (NCT00947167) meant to assess the activity of pertuzumab in association with erlotinib in a similar population, but this study concluded in early termination due to toxicity, thus the small number of patients analyzed (only 4) drastically reduced statistical power [61].

Crosstalk between ErbB and mTOR pathways might act as a potential resistance mechanism to targeted agents. Simultaneous blockade of both signaling yielded synergistic effects in cell models of several cancer entities and in nude mice. Synergistic cytotoxicity of erlotinib and everolimus have been observed also in lung carcinoid cell lines through a downregulation of EGFR/AKT/mTOR pathway, that seems to be activated in a consistent proportion of bronchopulmonary NENs [166]. Based on these findings, a phase 2 trial (NCT00843531) evaluating the efficacy of everolimus plus erlotinib in patients with progressive moderately to well differentiated NENs is ongoing, but results are not available yet [61] (Table 2).

### ALK

The receptor protein tyrosine-kinase ALK (anaplastic lymphoma kinase) is involved in cell survival, proliferation and oncogenesis. Chromosomal rearrangements generating ALK-fusion proteins as well as ALK overexpression and mutations seems to be involved in many oncological diseases. The EML4-ALK fusion gene plays a fundamental role in about 5% of NSCLC acting as a driver mutation and a therapeutic biological target [167, 168]. Aberrant ALK expression has been evaluated also in bronchopulmonary NENs resulting extremely rare though slightly more frequent in high-grade lesions and advanced stages correlating with poorer prognosis. Furthermore, ALK expression seems apparently not associated with gene rearrangement, amplification or mutations. Therefore, NENs do not seem to be optimal candidates for kinase inhibitor treatment [169, 170].

Nevertheless, ALK rearrangement has been occasionally detected in patients with ACs that have been successfully treated with ALK-inhibitor crizotinib [171–174]. To date, the role of ALK as a potential therapeutic target also in lung carcinoids is based exclusively on these case reports; furthermore, in all these cases gene rearrangement has been evaluated in biopsy specimens that might not be representative of the whole tumor.

Although these limitations, a basket study (STARTRK-2) is ongoing evaluating the role of entrectinib, a selective tyrosine-kinase inhibitor, in patients with solid tumors (included advanced NENs) harboring NTRK 1/2/3, ROS1 or ALK rearrangements (NCT02568267) [61] (Table 2).

### Other molecular targets

Analysis of neuroendocrine cell lines allowed to identify IGF-1 as a significant mediator involved in cell growth and peptides secretion mainly through interaction with AKT/mTOR pathway. Furthermore, IGF-1 receptors (IGF-1R) have been found at high concentration in NENs, thus blockade of this pathway might result in inhibition of growth and apoptosis [175]. A recent study analyzed genomic profile of 70 bronchopulmonary NENs specimens and IGF-1 expression was observed in 87.5% of TCs add 38.5% of ACs, suggesting a potential role of monoclonal anti-IGF-1R-antibodies (i.e. cixutumumab) in the management of well differentiated lung carcinoids [164]. On this basis, a phase 1 trial has been realized to evaluate the safety of cixutumumab in combination with everolimus and octreotide LAR in 19 patients with advanced well differentiated NENs of any site (including 4 lung carcinoids). Although promising preclinical results, this combination showed an unacceptable long term safety profile without a clear signal of activity [176]. Further studies are needed to identify potential predictive biomarkers and to evaluate cixutumumab in specific subset with more homogeneous population.

The novel CDK (cyclin-dependent kinase) 4/6 inhibitors represent a very promising therapeutic strategy in neoplastic diseases acting through downregulation of cyclinD-CDK4/6-Rb axis with cell cycle arrest in early phases. Several studies investigated the role of CDK 4/6 inhibitors alone or in combination with targeted agents, chemotherapy or PRRT on neuroendocrine cell lines with encouraging results [91, 177, 178]. Accordingly, two phase 2 trials are ongoing evaluating activity of ribociclib alone (NCT02420691) or in combination with everolimus (NCT03070301) in patients with foregut advanced NENs [61] (Table 2).

Mutations of chromatin remodeling genes represent the most important genomic aberration in lung carcinoids appearing in up to 73% of cases. Among these genes, MEN1 seems to act as a major player in the pathogenesis of well differentiated bronchopulmonary NENs with a significant role in their diagnosis [86, 179]. MEN1 pathway appears frequently inactivated through several mechanisms: gene mutations, allelic losses of MEN1 locus (11q13) or reduced activity of mutated menin [86]. MEN-1 pathway aberrations were also related to shorter survival in lung carcinoid patients showing also a prognostic value [13]. Unlike poorly differentiated neoplasms, mutations of cell cycle regulation genes (such as TP53 and RB1) are rarely identified in well differentiated NENs harboring a very low mutational burden [9]. In the era of immunotherapy and precision medicine, several studies focalized on antitumor inflammation status and PD-L1 expression in lung carcinoids. Based on a very low intratumoral CD8 + cell infiltratation, well differentiated thoracic NENs might be defined “immune deserted” with almost absent PD-L1 tumor expression [180, 181]. Nevertheless, many trials are currently ongoing evaluating immunotherapy alone or in combination with chemotherapeutic agents in mixed population of advanced NENs including bronchopulmonary carcinoids [61] (Table 2).

## Conclusions

Despite their indolent behavior, lung carcinoids correlate with decreased survival and are commonly insensitive to standard chemotherapy. To date, only limited therapeutic options are available and novel drugs are strongly needed in the management of advanced bronchopulmonary carcinoids. The rarity of the disease together with the cost of the available technologies (especially NGS) justify the lack of knowledge about the genetic landscape of carcinoid tumor compared to the most common SCLC [86]. Genomic aberrations appear to be also susceptible to intra-tumor heterogeneity, even in the same mass, and longitudinal heterogeneity with a considerable variability in the same patient over time [86, 182]. Furthermore, genomic aberrations seem to be relatively rare in lung carcinoid even when researched in completely resected tumors [Lou et al. 2016]. Further studies are necessary to identify new potential “druggable” molecular targets in the selected subset of low-grade lung carcinoids.

## Data Availability

Not applicable.

## Abbreviations

NENs: neuroendocrine neoplasm
pNENs: pancreatic neuroendocrine neoplasm
TCs: typical carcinoids
ACs: atypical carcinoids
SCLC: small cell lung cancer
LCNEC: large cell neuroendocrine carcinoma
NGS: next generation sequencing
PTH: parathyroid hormone
SSAs: somatostatin analogs
PRRT: peptide receptor radionuclide therapy
GEP: gastroenteropancreatic
IHC: immunohistochemical
SRS: octreotide scintigraphy
GH: growth hormone
IGF-1: insulin-like growth factor-1
LAR: long acting release
TTP: time to tumor progression
HR: hazard ratio
OS: overall survival
PFS: progression free survival
NR: not reached
ORR: overall response rate
VEGF: vascular endothelial growth factor
DAs: dopamine agonists
DR2: dopamine receptor 2
DOTA: tetraazacyclododecane-tetra-acetic acid
DTPA: diethylenetriamine penta-acetic acid
PCR: polymerase chain reaction
WB: western blot
DCR: disease control rate
FDG: fludeoxyglucose
TTF-1: thyroid transcription factor 1
SUV: standardized uptake value
5-FU: 5-fluorouracil
DLT: dose limiting toxicity
mTOR: mammalian target of rapamycin
GLUT-1: glucose transporter 1
4E-BP1: eukaryotic initiation factor 4E binding protein-1
p70S6K: protein S6 kinase
mTORC1: mTOR complex 1
IRS-1: insulin receptor substrate-1
PI3K: phosphatidylinositol 3-kinases
Akt: protein kinase B
MAPK: mitogen activated protein kinase
PR: partial response
MGMT: methylguanine DNA methyltransferase
CAPTEM: capecitabine and temozolomide
SD: stable disease
XELOX: oxaliplatin and capecitabine
GEMOX: gemcitabina and oxaliplatin
PEG-IFN: pegylated interferon
IFN-α-2b: interferon alfa-2b
TKI: tyrosine-kinase inhibitor
GIST: gastrointestinal stromal tumor
CBR: clinical benefit rate
EGFR: epidermal growth factor receptor
NSCLC: non small cell lung cancer
CR: complete response
ALK: anaplastic lymphoma kinase
IGF-1R: IGF-1 receptors
CDK: cyclin-dependent kinase
